# A critique of the use of species and below-species taxonomic terms for viruses—time for change?

**DOI:** 10.1093/ve/veae096

**Published:** 2024-11-22

**Authors:** Peter Simmonds

**Affiliations:** Nuffield Department of Medicine, Peter Medawar Building for Pathogen Research, University of Oxford, Oxford United Kingdom

**Keywords:** taxon, scientific name, species, synonym, subspecies, virus nomenclature

## Abstract

The International Committee for the Taxonomy of Viruses (ICTV) regulates assignment and names of virus species and higher taxa through its taxonomy proposal and ratification process. Despite using similar taxonomic ranks to those used elsewhere in biology, the ICTV has maintained the principle that species and other taxa are strictly categories with a formal nomenclature, whereas the viruses as objects are referenced through a parallel inventory of community-assigned virus names. This is strikingly different from common and scientific name synonyms for species used elsewhere in biology. The recent introduction of binomial names for virus species resembling biological scientific names has intensified this confusion in terms within the virology community and beyond. The ICTV taxonomy furthermore does not engage with or regulate classification below species and consequently lacks taxonomic terms or descriptions for important viral pathogens such as polioviruses, severe acute respiratory syndrome coronavirus type 2, HIV-1, and avian influenza as examples. The consequent reliance on community-adopted virus names, genotypes, and other categories often lacks clarity for clinical, biocontainment, and other regulatory purposes. This article proposes a revision of rules and procedures for species and below-species level classification. It recasts virus and virus species names as ‘common’ and ‘scientific’ names that are used in other biology nomenclature codes, each with expanded reference to both object and taxon. It further advocates the creation of a formal below-species taxonomic rank to define a new inventory of approved taxa and specified nomenclature below species. Adoption of the proposed changes will realign virus taxonomy with other biological nomenclatural codes and provide greater transparency and clarity in virology, medical, and regulatory fields.

## Introduction

The following review describes the principles of nomenclature for viruses and the species to which they are assigned. This proposes changes to the current reference of virus and species names to objects and categories, respectively, and the extension of virus taxonomy below the rank of species. Although these areas have been under discussion within and outside the International Committee for the Taxonomy of Viruses (ICTV) for many years, this review and the drafting of proposals for change have been precipitated by the effects of the recent change of virus species names to a binomial format and the consequences this may have for the usage of virus and taxonomic terms by the virology community (Siddell et al. 2020, 2023).

Box 1. **What is the problem?**

The following exchange between the author (P.S.), a member of the ICTV Executive Committee, and an experienced virology principal investigator (PI) exemplifies why future changes in the reference of terms used for virus names and virus species might be considered. The discussion seemingly at cross-purposes from the start took place at a recent workshop on hepatitis C virus (HCV) in the UK immediately after a presentation by P.S. where the new way of naming virus species as Latinized binomials was described, using examples from the Flaviviridae family:

PI That was a useful update on virus taxonomy and very interesting to hear about the changes to the name of HCV by the ICTV.

P.S. Many thanks, although as I presented and tried to emphasize, it’s not the name of the virus that is being changed, it’s a change to the name of its species to the new Latinized binomial form.

PI Yes, it’s very interesting to see these Latin names being used, but I wonder how long it will take clinicians to get used to using, what was it, *Hepacivirus*, er … hominis?

P.S. Well, the clinician would still refer to it as HCV….

PI … but we now know that its real scientific name is now going to be *Hepacivirus hominis* … how about we abbreviate it to HHV possibly?

P.S. < inwardly groans, deep breaths> I enjoyed your student’s presentation too ….

P.S. thought he had presented the new conventions for naming virus species quite clearly—as advocated for and introduced by the ICTV (Zerbini et al. 2022), these change were designed as a step toward bringing virus taxonomy closer to practice elsewhere in biology and were intended to provide greater clarity to the concept of virus species as taxa. The concept and reasoning behind it was obviously more difficult to communicate than P.S. had imagined.

Many readers may well be familiar with the type of exchange and incomprehension that surrounds attempts to use species and virus terms correctly, and indeed as recently reviewed, there is widespread misuse of both terms in the virology literature spanning three decades. I will attempt to explain why this might be the case.

The article describes the classification principles of viruses, bacteria, and archaea, particularly in relation to how species have been defined in the absence of sexual reproduction and the inapplicability of the biological species principle (Mayr 1942, Mayden 1997). As demonstrated, the frequent misconceptions about taxonomic terms used for viruses revolve around the various ways in which categories have been used in different areas of biology. How this incompatibility arose is described and a recasting of virus species terms to embrace the wider reference of scientific names used in mainstream biology is proposed.

## Background—virus classification

Under the wider banner of microbiology, viruses and bacteria are the primary causes of medical, veterinary, and agricultural infectious diseases. While bacteria and other cellular forms of life are believed to have originated from a common ancestor, viruses are now believed to have more recently originated independently multiple times (Brussow 2009, Krupovic et al. 2019, Nasir et al. 2020). Virus groups with evidence of a common origin have now each been assigned to separate realms (Kuhn et al. 2019, Koonin et al. 2020), the highest taxonomic rank in the expanded classification of viruses developed by the ICTV (Koonin et al. 2020). Classification within each realm is based upon the shared possession of evolutionarily related hallmark genes that demonstrate their common origin. Such lineage markers may be genome replication or virion formation genes, including genes for the capsid structures of members of the realms *Duplodnaviria* and *Varidnaviria*, or the RNA-dependent RNA or DNA polymerases of viruses of most RNA and reverse transcribing viruses in the realm *Riboviria*.

Below this level, viruses and prokaryotes are classified into similar taxonomic ranks, including kingdoms, phyla, classes, orders, families, genera, and species. Of these, assignments at the species rank serve most obviously as descriptions of agents of infectious diseases or inhabitants of particular ecological niches in the wider environment. Species names, typically in the form of genus + species epithets for prokaryotes, and recently introduced for viruses, is inherited from a system developed by Linnaeus in the 18th century for animals, plants, and fungi (Cain 1993). However, while species assignment has relied, in part, on a demonstrated or assumed capacity to interbreed and to consequently share membership of a common gene pool (Mayr 1942, Mayden 1997), this criterion cannot be used for the asexually reproducing organisms such as prokaryotes or viruses. Furthermore, in view of their microscopic size, until the second half of the 20th century, species assignments of bacteria tended to be primarily focused on the characteristics of their associated diseases and epidemiology rather than on the causative organisms This influence is still evident in current taxonomic nomenclature—tuberculosis is caused by bacteria of the species *Mycobacterium tuberculosis*, cholera by *Vibrio cholerae*, etc.

Historically, the assignment of virus species has been similarly matched to their associated diseases or virion morphology rather than being based on evolutionary relationships of the causative viruses. Thus, the agents of the distinct mosquito-borne diseases Japanese encephalitis, chikungunya, and dengue fever were assigned to separate virus species because of the distinguishable characteristics of the diseases they cause. As virus genome sequence acquisition accelerated through the 1980s, there was a transition from classifications based on disease or phenotypic properties of viruses to ones primarily defined by genetic relationships. Methodological advances, such as large-scale nucleotide sequencing of virus genomes and the exploitation of viral protein secondary structure comparisons to identify more distant evolutionary relationships (Sinclair et al. 2017, Mönttinen et al. 2021), have provided a wealth of new information on virus genetics and insights into their evolutionary relationships that were often largely lacking in their original phenotypically based classification.

Indeed, the ICTV now provides taxonomic assignments for viruses known only from the nucleotide sequences of their genomes, as long as there is evidence that this is at least coding complete (Simmonds et al. 2017). Based on this principle, large-scale virus sequence acquisition using high-throughput sequencing platforms (Roossinck 2012, Brum et al. 2015, Li et al. 2015, Shi et al. 2016, Käfer et al. 2019, Koonin et al. 2020, Wolf et al. 2020) has led to the discovery of thousands of novel viruses. As a consequence, the number of virus species has risen rapidly in recent years (>14 000 by 2024) with a likelihood of further substantial (10- or even 100-fold) expansion in the future. For most of these viruses, there is little information about their phenotypic or epidemiological properties (e.g. host range, geographical distributions, or possible disease associations), despite these being the features previously used for virus classification.

### Genetics-based species assignments

Although the ICTV requires that species assigned from virus sequences must form a single genetically distinct (monophyletic) lineage distinct from other classified species (Siddell et al. 2023), there is no prespecified threshold of nucleotide sequence identity that defines species. Patterns of natural genetic clustering often represent the primary metric available for their classification. Taxonomic assignments to genera, families, and higher ranks are similarly based on genetic relationships and principles of monophyly of hallmark genes, such as the RNA-directed RNA polymerase for RNA viruses (Wolf et al. 2018).

While the species taxon assignment is a basic requirement and building block in virus taxonomy, the substantial and intrinsic arbitrariness of assignment criteria means that classification at the species rank has none of the biological meaning that might be pinned to species of sexually reproducing organisms. There is, for example, no fixed relationship between virus species-level groupings with population structures or shared gene pools that are characteristic of sexually reproducing eukaryotes.

## What do species and virus names refer to?

### Names of organisms and taxa

Apart from the practical and organizational differences between the criteria used to assign species of viruses, prokaryotes, and other forms of cellular life, it is virus nomenclature and the reference to categories of virus species and other classification ranks that mark out the distinctiveness of virus taxonomy.

In their original formulation, species were devised to mark the finest division of animals and plants possible for biologists where interbreeding capability was typically used to define membership of the same or different species. Despite the many exceptions, inaccuracies, and frequent empirical difficulties of determining interbreeding capability, the so-defined species entry remains the fundamental basic unit in eukaryotic taxonomy. Genera, at the next taxonomic level, then approximated to the ‘type’ of organism—hence ‘generic’. For example, oak trees (genus *Quercus*) in Britain divide into two reproductively isolated native species [*Q. petraea* (sessile oak) and *Q. robur* (pedunculate oak)] that coexist with several imported *Quercus* species.

From the outset of the systematic classification of organisms, the Latinized binomial name (LBN) format of genus + species epithet was developed as a means to provide an internationally agreed scientific name and description for each defined species. Names were originally in Latin and thus independent of the multiplicity of language-specific terms; LBNs are thus a *lingua franca* for biologists, where *Q. petraea* might be termed ‘sessile oak’ in English, ‘Traubeneiche’ in German, ‘talvitammi’ in Finnish, and ‘dąb bezszypułkowy’ in Polish. At the outset (and relevant to the discussion below), a species name in the LBN format carries the same dual reference to an actual example of the species as would the ‘common’ name. *Quercus robur* could refer both to a physical example of the species (the oak tree at the end of the field) and to the set of cross-fertile and morphologically similar trees that extends from Northern Britain, continental Europe through Turkey to the Asian steppes. As indeed can the common name—‘The sessile oak is widely distributed in Europe and Western Asia’.

### The reference of virus and virus species terms

Following publication of the original species lists by Linnaeus (Cain 1993), conventions for species definitions and their reference to organisms and associated nomenclature using LBNs have been extended and refined for all types of cellular life, including fungi and other unicellular eukaryotes, bacteria, and archaea. Similar to its originally intended biological usage, microbiologists can use the binomial species name to refer to a taxonomic category (e.g. ‘there are many serogroups in the bacterial species *Neisseria meningitidis*’) as well as to an instance of a bacteria [e.g. the meningococcal serogroup B vaccine (4CMenB) prevents infection with *N. meningitidis*]. As with oak trees, a ‘common’ name, such as meningococcus for *N. meningitidis* or pneumococcus for *Streptococcus pneumoniae*, is also simply an English language synonym for the binomial name and can similarly also refer to both the species taxon and the actual organisms classified within the species.

Contrastingly, virus name and category usage do not follow the shared reference of species names to categories and objects. Since the 1990s, the ICTV has maintained a strict and vigorously enforced distinction in reference between the species (as a human-made category used for taxonomic classification) and its member viruses (as physical objects). This convention arose primarily from the original and highly influential treatise ‘Viruses are real, virus species are man-made, taxonomic constructions’ and subsequent writings by Marc van Regenmortel (Van Regenmortel 2003, 2007, 2016, 2018). Under this view, taxonomic terms such as species refer only to the human-made categories or classes defined in the ICTV taxonomy (Box 2). The members of these taxa are the actual viruses, i.e. they are objects rather than categories of objects. Thus, members of taxa have a physical and observable existence, e.g. as virions visualized by electron microscopy and as a manifestation of the cytopathology from replicating viruses within infected cells.

Box 2. **Objects and categories in biological and linguistic usage**

Marc van Regenmortel advanced the highly influential view that categories such as species require the formation of an abstract class with a membership of viruses assigned to it (Van Regenmortel 2003, 2007, 2016, 2018). This restricts the reference of a species and other virus taxonomic terms to the category. In parallel, viruses as members of taxa would possess only object reference and would carry separate names and typographical differences from the names of species and other taxa to which they were assigned. The need to uphold this distinction in reference was not uncontested at the time (Bos 2000, 2003, Gibbs 2003, Gibbs and Gibbs 2006); Bos, for example, had argued that a part/whole relationship apparent in normal biological usage of the species term (such as the use of *Q. robur* to refer to the tree at the end of the field) should be applied to virus species too and conversely that language-specific terms for viruses might also possess category reference (Bos 2003). Gibbs proposed greater congruence of virus taxonomy with other biological codes, such as the use of language-specific ‘common names’ and a separate and distinctive nomenclature for a taxonomically defined species each with a ‘type’ description (Gibbs 2003). Other biologists similarly found the concrete versus abstract distinction to be inappropriate in the radically different and widely used evolutionary paradigm that casts species simply as physical collections of a replicating lineage in a defined space and time. In this framework, species can only be described and assignments to them cannot be based on inclusion and exclusion criteria that are typically used to define a class or category (Claridge 2010, Mishler 2010).

Quite independently of the biological framework of species taxa, the distinction between a category and the entities assigned to that category is also quite inconsistent with common language usage. As a simple standard example (Hey 2001), the Earth may be classified as an instance of the astronomical category ‘planet’, as might Mars, Jupiter, and others in our solar system, planets being defined as being typically large, round in shape, and in a stable orbit around a star. This definition differentiates planets from other celestial entities, such as comets, asteroids, and moons. However, it is also possible to create sentences such as ‘a meteorite crashed into the planet’ or ‘the planet toppled into the fiery sun’, where ‘planet’ refers to a physical object—an instance where a member of a category takes on the terminology and reference of the category to which it is assigned.

For the majority of virologists, the typological distinction rarely impinges on standard virus name usage or on their classification. However, the category and object distinction enforced by the ICTV has created severe and often seemingly inexplicable complexities and restrictions in virus and species nomenclature that is absent elsewhere in biology. As a manifestation of this, the phrase ‘my daughter was infected with measles virus’ refers to the virus as physical agents of infection, but the phrase ‘my daughter was infected with *Morbillivirus hominis*’ is regarded by the ICTV as incorrect, the argument being that you cannot be infected by an abstract taxonomic category, only by a physical member of that abstract taxonomic category.

Unlike sessile oaks and *Q. petraea*, this restriction flies in the face of long-established conventions used elsewhere in biology and indeed for the use of categories in the wider cognitive and linguistic context (Box 2). While common terms such as the English language ‘sessile oak’ and the scientific name *Q. petraea* are regarded as synonyms, the ICTV specifies that a second set of largely unregulated names, often with local language variants, is used for viruses as objects. This runs in parallel to the ICTV-regulated nomenclature for the species taxa to which viruses are assigned.

The ICTV has recently mandated the use of binomial names for all current and future virus species (https://ictv.global/filebrowser/download/4902). The use of a binomial format was deliberately designed to make virus species names more obviously recognizable and compatible with the rest of biology. However, the ICTV conceived of binomial names as a means to actually reinforce a distinction between category and object, rather than the change toward a system of scientific names and common (virus) names used elsewhere in biology. Unfortunately, and perhaps not surprisingly in retrospect, their adoption of binomial names for virus species seems to have actually increased rather than reduced terminological confusion, as exemplified by the conversation in Box 1. If one mistakenly assumes that *Hepacivirus hominis* now denotes the scientific name of HCV, then surely patients can be infected with *H. hominis* in the same wame way that they could be infecrted with *Escherischia coli*. The resemblance of terms used in microbiology would similarly perplex an infectious doseases doctor, who might diagnose a patient with *Streptococcus pneumoniae* but might then not understand why, under ICTV taxonomy rules, the patient cannot also be infected with *Respirovirus pneumoniae*. In the latter case of a virus infection, the correct terminology would be that the patient was infected with a member of the species *Respirovirus pneumoniae* or using the virus name, infected with human parainfluenza virus 3. With binomial name formats resembling those used in microbiology, but with different underlying references to categories and objects, incorrect usage of virus species names will likely become far more widespread than before.

## Below-species classification

Bacterial and virus species classifications were originally based on descriptions of observable properties of the infectious agents, such as disease manifestations of infections or the host range and geography of the infectious agent. However, these assignments show quite varied associations with metrics of genetic relatedness of the causative agents that are now more widely used for classification, at least in virology. As described earlier, where one places a genetic threshold for species (and higher ranks) is highly variable, particularly between virus groups, and ultimately somewhat arbitrary. Consequently, adequate descriptions and classifications of viruses often extend (outside the remit of the ICTV) below the species rank, these often being based on possession of distinct serological and cross-neutralization properties (serotypes), patterns of genetic clustering (genotypes or lineages), or pathogenicity.

Below-species classifications of viruses do not, however, lead to the creation of formal subspecies categories since species is the lowest rank recognized by the ICTV. This practice is distinct from subspecies classification in zoology where trinomial names can be assigned, for example, to geographically distinct populations that can nevertheless interbreed (e.g. *Pan troglodytes verus* and *P. t. troglodytes* for West and central African chimpanzees). Botanical classification can involve a much wider range of named taxonomic divisions, such as subspecies (e.g. *Poa secunda* subsp. *juncifolia*), varieties (e.g. *Acanthocalycium klimpelianum* var. *macranthum*), cultivars (e.g. *Pinus nigra* ‘Arnold Sentinel’), or even two lower rank levels of form and cultivar (e.g. passion fruit—*Passiflora edulis* f. *flavicarpa* ‘FB200’). These additional designations below the level of species lead to the creation of formal taxonomic names with associated published descriptions for them. They also encapsulate the same dual reference to categories and component organisms as used in species assignments.

Because assignments and nomenclature of viruses to groupings below the species rank carry no formal taxonomic status, the unregulated nomenclature consequently refers only to organisms rather than taxa. This fits poorly for viruses assigned to relatively broad virus species categories. Indeed, this grouping together of quite dissimilar viruses greatly reduces the value of their species assignment as a description of their properties and evolutionary relatedness. An example where this becomes problematic concerns poliovirus types 1-3 that are not taxonomically differentiated from the largely nonpathogenic coxsackievirus (CV) types (e.g. serotypes CVA20, CVA22, and CVA24) since they are all classified into the same species *Enterovirus coxsackiepol*. Similarly, distinct strains of noroviruses, such as Lordsdale virus, Maryland virus, Jena virus, Alphatron virus, murine norovirus 1, Viseu virus, dog norovirus, Chiba-040502 virus, Sapporo-HK299 virus, and bat norovirus, are all assigned to the species *Norovirus norwalkense*. Similarly, human-infecting hepatitis E virus (HEV) and related viruses infect pigs, wild boars, rabbits, and camels in the species are assigned to the species *Paslahepevirus balayani*. In these and comparable cases, the unregulated virus name may actually be the more informative label than its species assignment, even though it is not described or defined as a taxon by the ICTV. Using one of the above examples, the statement that patient X is infected with a member of *E. coxsackiepol* is inadequate as a clinical description, when compared to one that differentiates a wild-type poliovirus type 3 infection (an international public health disaster) from those by CVA22 or CVA24, for which there is no evident clinical relevance.

There is the additional inconsistency that below-species nomenclature possesses both object (virus) and category reference, with terms such as types, isolates, and variants sharing names with the viruses assigned to them. For example, HCV (species *Hepacivirus hominis*) is relatively diverse genetically with eight genotypes currently assigned using a 32%–33% nucleotide sequence divergence threshold. However, this below-species level classification, in wide clinical use, uses the same genotype terminology for categories, such as in ‘Variants infecting injecting drug users in the UK are largely of genotypes 1a and 3a’, and for viruses as objects, such as ‘the patient in the ward is infected with genotype 1a’. This is perfectly clear linguistically (rather like the ‘planet’ in Box 2), where the context is used to determine whether the term refers to a category or an object. However, this is confusingly inconsistent with the ICTV position that a separate nomenclature is required for objects and categories.

In view of the frequent importance of virus groupings below the rank of species as clinical or epidemiological entities, members of many of the current ICTV Study Groups already take on responsibility for type, genotype, serotype, strain, or other subspecies classifications and nomenclature in consensus recommendations (as examples, Simmonds et al. 2005, 2020, Fauquet et al. 2008, Kuhn et al. 2010, Smith et al. 2014) and websites (e.g. https://picornaviridae.com; https://ictv.global/sg_wiki/flaviviridae/hepacivirus) that provide guidance for usage by the virology community. These activities are a fairly seamless extension of the role of these Study Groups in developing virus taxonomy, even though the subspecies groupings are unrecorded by the ICTV and possess no formal taxonomic status.

### Principles of nomenclature

The names of cellular organisms as objects may be based on the name of the species to which it is assigned, as a scientific name, or may bear a common, often language-specific name as a synonym. However, virus names are purposefully different from their species assignments, and rather like common names elsewhere in biology, are entirely unregulated and unrecorded by the ICTV, being formally outside of its remit. Virus names may take a variety of forms, with often separate names in different languages, such as Masern Virus, virus de la rougeole, morbillo virus, and tuhkarokko virus in German, French, Italian, and Finnish, respectively, for measles virus in English (species *M. hominis*). There may also be multiple names in one language for the same virus, such as black bullhead herpesvirus and ictalurid herpesvirus 2, and bovine alphaherpesvirus 1 and infectious bovine rhinotracheitis virus, again analogous to the use of multiple terms used elsewhere in biology such as ‘European oak’ and ‘common oak’ for *Q. robur*.

This variability in virus names is not intrinsically problematic as their use is defined by their attribution of a species and resembles the usage of common names elsewhere in biology. The problems arise when such nonstandardized and often undefined names are used to refer to groups of viruses that are not assigned taxonomically. For example, HEV was originally identified as a cause of human enterically transmitted hepatitis, but the same name is now also used to refer to highly divergent viruses among many species in the family *Hepeviridae* that infect bats, rats, pigs, and a range of other mammalian hosts. The term HEV could be regarded as inappropriate for this extended range of viruses that probably do not cause hepatitis or are even necessarily hepatotropic. Indeed, in various forms, the term ‘hepatitis E virus’ seems to have been coined in the names of all viruses assigned to the genetically highly diverse subfamily *Orthohepevirinae*. The differentiation of viruses within the subfamily using a host prefix like ‘bat’ serves little taxonomic purpose when bat-infecting HEVs may be found in more than one species. Virus terms in other families, such as ‘bat hepacivirus’, ‘bat circovirus’, and similar rat prefixes for species of pestiviruses, circoviruses, and hepaciviruses similarly each refer to members of multiple species. Viruses assigned to small DNA virus families such as *Circoviridae* and *Smacoviridae* have multiple collisions of names.

As the pace of virus discovery and genetic characterization increases, these virus nomenclature inconsistencies and gaps may widen considerably even if the ICTV manages to keep up with their classification and taxon nomenclature. If virus taxonomy expands in the next decade to include over 100 000 species, this would seemingly require the virology community to come up with an equal or larger list of corresponding virus names for members of each of these species, and perhaps for variants within some of these. This is a problem somewhat unique to virology; dual nomenclature is largely absent and considered unnecessary in bacterial taxonomy and elsewhere in biology which, beyond familiar macroscopic organisms, primarily use scientific names with combined category and object reference.

### Regulation of virus nomenclature

There are many areas where clarity and precision in virus names is essential—for example, in the formulation of import and export regulations of plants and livestock to exclude viruses, such as foot-and-mouth-disease virus. Clear reference to a standardized virus name is also required for the assignment of biosafety levels (BSLs) and containment regulations for working with pathogenic viruses. While this is an aspiration, actual practice is less than ideal; for example, clear reference and categorizations of virus groups are seriously lacking in many official regulatory documents such as the current UK Advisory Committee for Dangerous Pathogens (ACDP) Approved list (5th Edition, published 2023) (Anonymous 2023) and the recently published inventory of ‘Priority Pathogens’ by the World Health Organization (WHO) (Anonymous 2024). In contrast to the unambiguous listing of bacterial, fungal, and other eukaryotic pathogens through the use of their scientific names and where necessary subspecies or strain names, most entries for viruses are a confusing mess of virus names, nonitalicized species or genus names, hybrid species, and virus terms such as *Mastadenovirus blackbeardi*-14 and similar category mistakes that are often of highly questionable clarity. For example, the entry ‘Orthohepevirus A (formerly known as Hepatitis E virus)’ in the ACDP list possesses an incorrect species name (it should be italicized and is in any case superseded by the species name *Paslahepevirus balayani*). The entry also makes the incorrect statement that the virus name ‘Hepatitis E virus’ has been replaced by the species name, and finally the scope of the virus name is unclear—does ‘hepatitis E virus’ refer to all members of the species or just the human viruses, or conceivably all members of the entire subfamily of *Hepeviridae* that incorporate ‘hepatitis E virus’ in their virus names? A simple and easily implementable fix would be to always use the species name along with a below-species rank where this is applicable—but current ICTV rules forbid both the use of a taxonomic name to describe viruses in the ACDP list (because they are physical objects), and there is no official nomenclature for viruses classified below the level of species.

Similar incorrect categorization and nomenclature are found in the ACDPs listing of human enteroviruses (Table 1). For example, the entry ‘Enterovirus D, Human Enterovirus type 70, Synonyms: Coxsackievirus CA24 (A24); Enterovirus 70’ includes an incorrect and wrongly formatted species name and a list of member viruses that incorrectly excludes EV-D68 and at the same time incorrectly includes CVA-24, a virus that is assigned to a different enterovirus species. Equating poliovirus with an incorrect species name as in ‘Human enterovirus C type 3 (also known as Poliovirus)’ is a potentially dangerous misnaming given the existence of multiple nonpoliovirus types in the *E. coxsackiepol* with different handling restrictions.

**Table 1.** Example of erroneous virus nomenclature in the ACDP report^a^: for human enteroviruses

Biological agent	HPHG^b^	Taxonomy/notes
Enterovirus D, Human Enterovirus type 70	2	Synonyms: Coxsackievirus CA24 (A24); Enterovirus 70
Human Enterovirus A also known as Coxsackieviruses (A)	2	
Enterovirus B (which includes the subspecies Echoviruses and the coxsackieviruses)	2	
Human enterovirus C type 1 (also known as Poliovirus)	2	Poliovirus vaccine available
Human enterovirus C type 2 (also known as Poliovirus)	3	
Human enterovirus C type 3 (also known as Poliovirus)	2	

a https://www.hse.gov.uk/pubns/misc208.pdf (Anonymous 2023).

b Proposed containment level—human pathogen hazard group.

Similarly, other regulatory lists, such as the Biosafety in Microbiological and Biomedical Laboratories document from the US Centers for Disease Control (Anonymous 2020) largely omit references to virus species taxa and are largely based on informal virus names, nontaxonomic terms such as arboviruses and a frequent dependence on over-broad virus family rank divisions. It is clear that something is very wrong with the communication of virus nomenclature under the current ICTV framework.

## Does virus nomenclature and category reference need to change?

This article highlights some of the problems associated with the reference of virus taxon and object terms and their distinct nomenclatures specified by the ICTV, with the exclusion of below-species taxonomy. Comparisons with practice elsewhere in biology highlight many important differences in their underlying frameworks and identify areas where greater cross-disciplinary understanding and harmonization of terminology would be of value. Of the many issues raised in the review, the following are examples of practical questions that a future revision of the virus taxonomy framework might seek to address:

- The adoption of a common taxonomic framework for zoology, botany, and mycology has been also effective for the classification of bacteria, archaea, and other asexual organisms such as unicellular eukaryotes without assistance from the biological species principle. Given this framework, why does the ICTV use a different approach with distinct terms for category and object reference instead of the simple ‘common’ and ‘scientific’ name terminology used elsewhere?
- The adoption of different rules and nomenclature for virus and taxon entities does not seem to acknowledge normal language usage. If ‘planet’ (or indeed virtually any other noun in any human language) can semantically possess both category and object reference (Box 2), why are virus and species terms uniquely differentiated by the ICTV?
- Given that species assignment thresholds are somewhat arbitrary, why does the ICTV insist on a terminological distinction between taxon and virus at the species level, when dual reference is permitted and works quite satisfactorily for genotypes, serotypes, or other below-species groupings?
- Certain virus entities, such as HIV-1, severe acute respiratory syndrome coronavirus type 2 (SARS-CoV-2), polioviruses, and enterovirus D68 in distinction from other enteroviruses, have important regulatory and biocontainment restrictions that differ from other members of the species to which they are assigned. In situations where clarity in their descriptions, definitions and nomenclature are absolutely essential, why does the ICTV, as the primary internationally recognized agency for virus classification, not engage itself with this area?
- Similarly, the use of different virus names in non-English languages is quite incompatible with the drafting of international regulations for containment and trade, and hinders communication of clinical, veterinary, and agricultural virus disease descriptions in different languages. The use of the standardized binomial species name (which would then be applied to the objects) and the extension of the ICTV remit to sub-specific ranks would solve this particular problem.
- The case definition and use of the term COVID-19 disease term to describe disease responsible for the 2020–22 coronavirus pandemic was an essential step by the WHO. In parallel, the *Coronaviridae* Study Group coined the term SARS-CoV-2 for the causative agent (Gorbalenya et al. 2020) and the virus name now universally used. However, neither the name nor its taxonomic assignment falls within the formal remit of the Study Group or wider ICTV. Indeed, its classification simply as a member of the species *Betacoronavirus pandemicum* fails to distinguish it taxonomically from other members of the species, such as SARS-CoV and a range of bat coronaviruses. While the Study Group was obviouslyconstrained by current ICTV rules, was this an appropriate and adequate response? How can medically important viruses be officially classified when they are only distinguishable from other viruses below the species level? There appears to be no official body or bodies to define and maintain such lists for the virus or wider community, despite its importance for infectious disease clinicians and veterinarians, public health, and for pandemic preparedness.

A possible review of the current restrictions in the use of virus and virus species names as objects or categories has not been under active discussion within the ICTV since the extensive controversies 20 years ago (Bos 2003, Gibbs 2003, Van Regenmortel 2003) when the current distinctions in reference were established. Indeed, the area may seem an obscure and marginally relevant topic for virological discussion, but I would argue that there is a pressing case for re-evaluating the current taxonomy framework and its scope of operation. In the next section, I describe how existing species names might be recast as scientific names as they are in the rest of biology. Extension of virus classification below the level of species is also proposed for viruses where this is required for scientific, clinical, and regulatory purposes. These proposed changes will address many of the current uncertainties and ambiguities in virus classification and virus taxon nomenclature. Collectively, these changes might be considered as the basis for a substantive future revision of the International Code of Virus Classification and Nomenclature (ICVCN) and future taxonomic practice.

## Specific proposals for changes to name reference and range

The following proposals and examples of usage described below are predicated by a fundamental change in name reference that allows viruses to be referred to directly by their species (or scientific) name (genus + species epithet and optionally a below-species assignment). Species names and virus names will possess both category and object reference, in common with usage in microbiology and wider biology.

The proposal also provides for the formal introduction of a below-species taxonomic level comparable to those used for subspecies in the taxonomies of cellular organisms. Categories used for below-species classification might be indicated by one of a list of approved designators that reproduce previously established terms such as ‘type’, ‘genotype’, ‘serotype’, or ‘isolate’, so as to minimize disruption to existing classifications. A ‘subspecies’ (ssp.) designator might also be used.

The following examples use a designator word followed by an italicized freeform below-species name. This represents one possible approach for naming below-species taxa. However, the actual nomenclature and typographic rules will have to be more widely debated and subsequently formally defined in an updated ICVCN code in the event that the ICTV agrees to incorporate below-species assignments in its taxonomy framework.

Box 3. **Proposals for changes to virus species and below-species assignment rules**

1. Names of taxa at the species level
  - Species (or scientific) names will correspond to the current ICTV binomial taxon names for species and have defined reference and prescribed name formatting.
  - Species names for viruses will have both category and object reference.
  - Species names will be extended to incorporate categories below the level of species where relevant (as described below).
  - The genus part of the binomial name can be abbreviated to the initial letter after first usage.
2. Common virus names
  - Some (but not all) virus names play a similar role to ‘common name’ conventions of other organisms. These represent names that have a one-to-one relationship with the taxon to which they are assigned. These should be strictly differentiated from below-species terms (described in section (3) of this box).
  - Common virus names should remain unregulated by the ICTV and may have alternative non-English language terms. They simply represent preferred or colloquial usage by the international virus community and wider public.
  - The use of a virus name must however be equated to and its reference defined by a scientific name on first usage in a document.
  - Common virus names must not be used in regulatory or other documents where precise reference is required—scientific names, including below-species assignments where these are necessary, should be used.
3. Below-species assignments and nomenclature
  - Below-species assignments and nomenclature should be part of the remit of the ICTV.
  - Virus variants can be assigned to a single below-species rank.
  - Some existing virus names may be repurposed in the nomenclature of below-species taxa.
  - The below-species designations will form a third part (suffix) to the species/scientific name of the species.
  - Several approved designator terms to describe below-species taxa will be available, and may include subspecies (ssp.), type, genotype, serotype, isolate, and potentially others.
  - Names used for below-species categories will be written in italicized type, comprise letters only from the Latin alphabet and numbers but without diacritics or accents.
  - Below-species taxon names should not be abbreviated after first use.
  - The regulation and updating of below-species assignments will be managed by the relevant ICTV Study Group.
  - Changes and additions should be actively solicited from the wider virus community and carry its support. Typically, below-species classification should match existing virus/type/strain assignments, nomenclature, and usage to minimize disruption to pre-existing classifications.

### Examples of proposed use

The following examples follow the format proposed in the three sections of Box 3, along with explanatory text within parentheses:

1. Taxon/scientific names
  - Measles virus (*Morbillivirus hominis*) widely infects nonimmunized children in many parts of the UK. (*This defines the reference of the term ‘measles virus’ to a classified taxon, and demonstrates the use of both common and scientific terms to refer to an object)*
  - Many species of hepaciviruses are known to infect the liver, e.g. *Hepacivirus hominis* and *H. equus* (*This statement adopts the convention from biology that species terms can refer to objects. It also exemplifies how an abbreviation of the genus name can be used after first use following the convention used elsewhere in biology*).
2. Common virus names
  - Outbreaks of yellow fever virus and dengue virus type 4 (*Orthoflavivirus flavi* and *O. dengue* type *dengue 4*) have recently erupted in Cuba *(This is an example of species and below-species variant designations [Orthoflavivirus flavi and O. dengue type dengue 4], and the use of both virus [common] and species [scientific] names to refer to objects)*.
  - Variants of human hepatitis E virus (HHEV; *Paslahepevirus balayani* genotypes *HEV1*, *HEV2*, *HEV3*, *HEV4*) ) are genetically quite divergent from rat hepeviruses (RHEV; *Rocahepevirus ratti*). (*Names and abbreviations that differ from standard usage can be coined if they possess clear reference to taxon names*).
  - Cette éruption cutanée est causée par le virus de la rougeole (*Mobillivirus. hominis*) (*This shows the use of a language-specific common name that is understandable and standard in French and which is clearly defined through its reference to the species’ scientific name. The ICTV therefore does not need to keep an inventory of language-specific terms for virus names)*.
  - For the purposes of this study, human zoonotic strains of hepatitis E virus (hHEV) are differentiated from those naturally infecting pigs (pHEV), all formally classified as *P. balayani* genotype *HEV3 (Terms are again nonstandard and these examples cut across taxonomic boundaries. Names, abbreviations, and scientific names are all used as categories)*.
3. Below-species nomenclature
  - Numerous recombinant strains of poliovirus 2 (PV-2; *Enterovirus coxsackipol* type *poliovirus 2*) circulate in sub-Saharan Africa (*This exemplifies the use of a below-species term as a category. The statement also incorporates a below-species taxon with a defined type designator derived from the virus name previously listed by the Picornaviridae Study Group*).
  - Bunyamwera virus [BuNV; *Orthobunyavirus bunyamweraense* ssp. *bunyamwera virus* is widely distributed in sub-Saharan Africa (*This is an example of a virus referred to as a subspecies within a wider species grouping that incorporates several other named viruses*).
  - The causative agent of the COVID-19 pandemic (*Betacoronavirus pandemicum* isolate *SARS Coronavirus 2*) is virologically and clinically quite distinct from SARS-CoV and other members of the species that infect bats (*This provides the much-needed formal taxonomic assignment for SARS-CoV-2 that is currently missing in the ICTV classification because of its current non-engagement with below-specific classification*).

## Impact of the use of revised taxon nomenclature

The changes proposed are designed to increase the clarity of reference of virus taxonomic terms, to create a nomenclature that better follows the usage of taxonomic terms used elsewhere in biology, and to provide a formally recorded terminology below the species level.

While these proposals represent a conceptual change in ICTV nomenclature policy, they represent a loosening of previous restrictions and add a taxonomic layer that was hitherto beyond its remit. Therefore, the new usage will not create incompatibilities with species references used in the existing virological literature. For example, a statement such as ‘Hepatitis C virus is a member of the species *Hepacivirus hominis*’ is fully supported under the new rules even though the reference of the scientific name has been extended. Similarly, poliovirus type 3 (PV-3) is still a member of *E. coxsackipol*, even though it can now be more precisely described taxonomically as *E. coxsackipol* type *poliovirus 3*. There would therefore be no terminological schism in the virological literature before and after their adoption.

The greater precision and range of taxonomic names may be used to update currently erroneous and poorly constructed regulatory documents, including the UK ACDP list of containment restrictions for microbiological pathogens (Table 2). As described in a previous section, the list of biological agents uses a complicated and often erroneous combination of virus names and virus species terms, leading to practical problems in using the document for laboratory management. The proposed substitution of species (or scientific) names (Table 2) immediately clarifies the reference of the virus terms to defined taxa at species and below-species levels. Its revised format becomes substantially similar to the species and subspecies terms used for bacterial, amoebic, and fungal pathogens listed in the same ACDP document.

**Table 2.** Proposed revised listing of enteroviruses in the ACDP report using new taxon terminology

Biological agent^a^	Common name	HPHG
*Enterovirus alphacoxsackie* ^b^	Enterovirus A (EV-A)^c^	2
*E. betacoxsackie* ^b^	EV-B	2
*E. coxsackipol* type *poliovirus 1*^d^	Poliovirus 1 (PV-1)	2 + GAP^e^
*E. coxsackipol* type *poliovirus 2*	PV-2	3 + GAP
*E. coxsackipol* type *poliovirus 3*	PV-3	2 + GAP
*E. coxsackipol* type *coxsackievirus A20*	CVA20	2
*E. coxsackipol* type *coxsackievirus A22*	CVA22	2
*E. coxsackipol* type *coxsackievirus A24*	CVA24	2
*E. deconjucti* type *enterovirus 68*^d^	EV-D68	3
*E. deconjucti* type *enterovirus 70*	EV-D70	2
*E. deconjucti* type *enterovirus D103*	EV-D103	2
*E. deconjucti* type *enterovirus D111*	EV-D111	2
*Cosavirus* spp.^f^	Human cosavirus	2

a The first column entries refer to viruses but are listed as scientific names.

b There are several serotypes classified in *E. alphacoxsackie* and *E. betacoxsackie*, but all are handled at the same containment level, so separate reference to them is not required for the list. For the purposes of the ACDP list, types need only be listed when handling restrictions differ.

c The species assignments are unambiguous, but the common names (such as widely used terms like EV-B and CVA22) can be referred to as synonyms without previous problems of trying to distinguish between category and virus reference.

d The separate listing of polioviruses and EV-D68 are examples where listing below-species level is required in situations where BSLs vary between types. Type classification in these circumstances therefore should fall within the remit of the ICTV.

e Assigned under WHO Global Action Plan (GAP) to minimize poliovirus facility-associated risk. Handling restricted to approved national reference laboratory facilities.

f This is an additional entry to the ACDP list to illustrate how the spp. suffix can be used to indicate multiple species within a genus.

Review of other regulatory lists, such as the Biosafety in Microbiological and Biomedical Laboratories document from the US Centers for Disease Control (Anonymous 2020) that are based only on virus community assigned virus names, nontaxonomic terms such as arboviruses and a frequent dependence on over-broad virus family level divisions, should be considered.

Since the reference of taxon names extends to viruses classified below the species level, a scientific name for a virus, such as *E. coxsackipol* type *poliovirus 1* would be entirely adequate as an annotation for GenBank and other INSDC records. This would avoid the current need for records to maintain both species assignment and virus names for each entry—a practice that is unnecessary and indeed absent in sequence records for other biological organisms.

As viruses classified below the level of species represent taxa, the Master Species List (MSL) maintained by the ICTV (https://ictv.global/msl) would therefore need to expand to include the range of designated genotypes, isolates, subspecies, etc. of species that will be formally assigned by the relevant Study Groups. This will require the additional data fields for designator and assigned names for taxa in below-species listings (schematic examples of entries are shown in Table 3).

**Table 3.** Proposed updated ICTV master species list format to include below-species taxon assignments and virus examples.

Genus	Species epithet	Designator^a^	Value^b^	Common name^c^	Abbreviation^c^	Examplar^d^
*Orthoflavivirus*	*flavis*	–	–	Yellow fever virus	YFV	X03700
*Hepacivirus*	*hominis*	Genotype	*HCV-1*	Hepatitis C virus type 1	HCV-1	AF009606
*Orthobunyavirus*	*catqueense*	Isolate	*Cat Que* ^e^	Cát Quếvirus	CQV	JQ675598; JQ675599; JQ675600
*Orthobunyavirus*	*catqueense*	Isolate	*Oya*	Oya virus	OYAV	JX983194; JX983193; JX983192

a This lists the designator type where an official below-species classification exists.

b Multiple entries replace the ‘Exemplar isolate’ and ‘Additional isolate’ listings in the existing Virus Metadata Resource (VMR)—broadly these serve the same purpose and represent instances where formal taxonomy assignments are distinct viruses within a species.

c Example virus names and abbreviation columns in the current VMR may be included in the MSL as under the current proposals, and taxon names can refer directly to viruses.

d INDSC accession number of exemplar virus.

e Non-Latin letters and diacritics are stripped from below-species names.

Regulation of below-species nomenclature should also come within the remit of the relevant ICTV Study Group. Additions and changes to virus classifications below the level of species will require submission of formal taxonomy proposals through the ICTV Executive Committee and ratification with oversight from the relevant Study Group. As most of the relevant classifications are already in place and widely standardized in the virus community and there is a choice of designators to match existing assignments, it would be relatively simple for Study Groups to formalize these into formal below-species taxonomic categories.

As previously discussed (Postler et al. 2017), the recent change of virus species names to a binomial format has resolved major compatibility problems for virus entries in biological databases where the two elements in the name are often by default assigned to genus and species fields (for example, by changing the previous species term *Measles virus* to *M. hominis*). The proposed creation of a formal below-species taxonomic level would enable virus assignments at this rank to be similarly incorporated into the subspecies field available in biological databases.

### Accountability

Since every relevant division of viruses will be represented as taxa, information on the criteria for assignment and their approval will be stored in and accessible from the MSL and associated taxonomic records held by the ICTV. Very much as currently recorded for species and higher rank taxa, these will provide definitive information on the approval process, including the criteria under which the taxon was defined and assigned, its nomenclature, dates of approval, and information on those involved in the decision. The existence of formal records will provide the basis for future modification and expansion of below-species taxonomy as this develops.

The development of formal recording contrasts markedly with the variable accessibility and publication records of currently described below-species taxa—some are described in consensus papers (e.g. Simmonds et al. 2005, 2020, Kuhn et al. 2010, Smith et al. 2014), some are listed on community websites (e.g. https://picornaviridae.com), while many represent traditionally used categories without available records of how their original assignments were made—e.g. dengue virus types 1–4.

### Flexibility in virus community usage

The existence of precisely defined scientific names for every classified virus will provide much greater clarity of reference that is independent of their common virus names. For example, as long as a virus name term is equated to a taxon at species or below-species level, its reference is unambiguous. This permits the use of language-specific virus names without the ICTV being required to maintain lists of these or impose English-language-only terms for viruses in below-species classifications, or indeed for species or higher rank assignments.

The advantages of this in terms of clarity and flexibility of use of common virus names are exemplified by the recent naming of SARS-CoV-2 as the causative agent of COVID-19. As described above, the importance associated with the naming of the causative virus in COVID-19 led the ICTV *Coronaviridae* Study Group to coin the virus name SARS-CoV-2. This classification and nomenclature decision was made at a below-species rank (SARS-CoV-2 is one of two human viruses interspersed with numerous bat virus lineages in the species *B. pandemicum*) and was therefore outside of the remit of the Study Group. The second problem is that the term SARS-CoV-2 was proposed for a virus rather than for a taxon—again outside the area of ICTV responsibilities. Third, its fixed format is somewhat inconsistent with the general principle that allows language-specific terms, particularly for clinically important viruses that are referred to outside of a strictly virology context. French clinicians, for example, use terms such as virus de l’hépatite B (VHB) for hepatitis B virus and virus de l’immunodéficience humaine de type 1 (VIH-1) for HIV-1 in their medical and other nonscientific literature, terms that similarly cannot be exclusively equated with a currently defined taxon. This lack of flexibility extends rather inconveniently for more informal literature where terms that writers may want to use terms such as the ‘COVID virus’ or ‘pandemic coronavirus’ that are undefined.

The adoption of a below-species taxonomy resolves all of these problems First, the definition and naming of SARS-CoV-2 as a subspecies taxon (i.e*. B. pandemicum* isolate *SARS Coronavirus 2*) would be within in the remit of the *Coronaviridae* Study Group. Its assignment as a formal, named taxon, and perhaps a parallel assignment and renaming of SARS-CoV to *B. pandemicum* isolate *SARS Coronavirus 1*, would provide scientific terms with defined and unique reference. French clinicians could write ‘Le patient a été infecté par le coronavirus du SRAS de type 2 (*Betacoronavirus pandemicum* isolate *SARS Coronavirus 2*)' unambiguously as could journalists—‘the COVID virus (referred to by scientists as *Betacoronavirus pandemicum* isolate *SARS Coronavirus 2*) spread into Italy in early 2020’ without having to use the SARS-CoV-2 name in the rest of the article.

## Conclusions

This article proposes an extension of the reference of virus taxonomic terms to objects and the extension of the virus taxonomy to include subspecies where these are required and appropriate. These key changes enable the creation of ‘scientific names’ to refer to viruses directly, very much as used for organisms elsewhere in biology. These suggested changes recognize that while there are unique challenges to virus taxonomy, there is nothing intrinsically problematic in areas of category and object reference of their taxonomic terms, nor classification below-species level that might justify current differences from taxonomic and nomenclatural codes in the rest of biology.

The proposed changes will bring clarity to virus taxonomy, solve many of the problems with virus nomenclature and reference, and create a transparent and formally recorded level of classification below species. As described, the proposals, if adopted, do not contradict or proscribe the current usage of species terms, and they support the continuing primary use of virus names to refer to the infections and diseases they cause. A compendium of approved taxonomic names for viruses classified below the species rank will hugely increase their clarity of reference in virology, medical, and regulatory fields.

## Data Availability

All data accessed in the article are publicly available through the cited sources.
